# Temperature-dependent modelling and spatial prediction reveal suitable geographical areas for deployment of two *Metarhizium anisopliae* isolates for *Tuta absoluta* management

**DOI:** 10.1038/s41598-021-02718-w

**Published:** 2021-12-02

**Authors:** Ayaovi Agbessenou, Komivi S. Akutse, Abdullahi A. Yusuf, Sospeter W. Wekesa, Fathiya M. Khamis

**Affiliations:** 1 International Centre of Insect Physiology and Ecology (Icipe), P.O. Box 30772-00100, Nairobi, Kenya; 2 Department of Zoology and Entomology, University of Pretoria, Private Bag X20, Hatfield, 0028 South Africa; 3 Forestry and Agricultural Biotechnology Institute (FABI), University of Pretoria, Private Bag X20, Hatfield, 0028 South Africa

**Keywords:** Biological techniques, Ecology, Microbiology, Zoology, Environmental sciences

## Abstract

*Tuta absoluta* is one of the most devastating pests of Solanaceae crops in Africa. We previously demonstrated the efficacy of *Metarhizium anisopliae* isolates ICIPE 18, ICIPE 20 and ICIPE 665 against adult *T. absoluta*. However, adequate strain selection and accurate spatial prediction are fundamental to optimize their efficacy and formulations before field deployment. This study therefore assessed the thermotolerance, conidial yield and virulence (between 15 and 35 °C) of these potent isolates. Over 90% of conidia germinated at 20, 25 and 30 °C while no germination occurred at 15 °C. Growth of the three isolates occurred at all temperatures, but was slower at 15, 33 and 35 °C as compared to 20, 25 and 30 °C. Optimum temperatures for mycelial growth and spore production were 30 and 25 °C, respectively. Furthermore, ICIPE 18 produced higher amount of spores than ICIPE 20 and ICIPE 665. The highest mortality occurred at 30 °C for all the three isolates, while the LT_50_ values of ICIPE 18 and ICIPE 20 were significantly lower at 25 and 30 °C compared to those of ICIPE 665. Subsequently, several nonlinear equations were fitted to the mortality data to model the virulence of ICIPE 18 and ICIPE 20 against adult *T. absoluta* using the Entomopathogenic Fungi Application (EPFA) software. Spatial prediction revealed suitable locations for ICIPE 18 and ICIPE 20 deployment against *T. absoluta* in Kenya, Tanzania and Uganda. Our findings suggest that ICIPE 18 and ICIPE 20 could be considered as effective candidate biopesticides for an improved *T*. *absoluta* management based on temperature and location-specific approach.

## Introduction

Tomato, *Solanum lycopersicum* L. is one of the most valuable cultivated vegetable crops in sub-Saharan Africa providing a source of direct and indirect employment for many people^1^. In Kenya, tomato is a food and nutrition security vegetable crop mostly cultivated by smallholder farmers for both domestic and export markets^2^. The rapid growth of the tomato industry has been coupled with the emergence of devastating indigenous and invasive insect pests and diseases^3^. As a result of these pests infestation, significant annual losses of up to 70% of tomato production have been estimated in Africa^3^, providing a clear constraint on current and future yields. Among these biotic constraints, the tomato leafminer, *Tuta absoluta* (Meyrick) (Lepidoptera: Gelechiidae) is ranked as the most current devastating pest of the crop^4,5^. *Tuta absoluta* is an invasive species native to South America^6^, which was first detected in 2008 on the African continent following a transatlantic invasion of tomato fields in 2006 in Spain^7,8^. More than a decade after its first detection in Africa, the pest has since spread to nearly every country on the continent destroying thousands of hectares of tomato fields and other solanaceous crops (e.g. potato, *Solanum tuberosum* L. and black nightshade, *Solanum nigrum* L.), frequently causing total crop losses^9^. The management of invasive pests on the African continent, especially for the tomato leafminer has taken a scary and reactive approach leading to sporadic and uncoordinated actions to control the pest which has over the years established a foothold in the continent^10^. The extent of damage and the associated alarming level of economic losses (estimated at US$ 1.1 billion) being reported annually due to *T. absoluta* are enormous and are likely to increase significantly if left uncontrolled, leading to additional production costs to manage the pest^3^.

In response to this challenging situation, smallholder vegetable farmers have been desperately applying cocktails of synthetic pesticides^11^, largely driven by government-subsidized agrochemical input schemes, aggressive marketing by pesticide company representatives and out of desperation to reduce the pest infestation. Yet, the widespread use of synthetic insecticides has rarely delivered a satisfactory level of control due to the cryptic feeding behavior of the pest immature stages and the rapid development of resistance to many classes of insecticides known for *T. absoluta* population in other parts of the world^6,12–14^, and consequently jeopardizing the pest control efforts. Additionally, pesticides resistance resulting from indiscriminate applications has caused unprecedented disruption of the resilience of natural ecosystems and has attracted growing public concerns over effects on non-target organisms, environmental and human health^15^. These resulted in the search for alternatives to pesticides to manage *T*. *absoluta* infestations with a great interest in developing biological control approaches using natural enemies (predators, parasitoids or microbials) to reduce pesticides use and limit insecticidal resistance development in both the adult and immature stages of *T. absoluta*^16–19^. Among the microbials being explored, entomopathogenic fungi (EPF) offer effective and viable alternative to control insect pests of economic importance, as they cause epizootics in the target host population while minimizing impacts on beneficial and other non-target organisms as well as increasing the quality of agricultural products^20,21^. These make them also potential option as biopesticides for controlling the tomato leafminer, *T. absoluta*^18^.

The mode of action of EPF against insects starts with spore adhesion to the host, followed by formation of appressoria that penetrate the cuticle, which later reach and invade the hemocoel and finally interferes with the host immune system^22^. However, the level of infection depends on the physiological properties (virulence, sporulation and persistence) of fungal strains which are regarded as a major obstacle to the success of their development as biocontrol agents^23,24^. Also under natural conditions, fungal infection is increasingly associated with stressful abiotic factors such as UV, humidity and temperature which modulate the virulence of the pathogen^25^. Indeed, there is increasing evidence that temperature is the dominant abiotic factor that has a significant influence on the infectivity profiles of fungal strains^26–29^ whose application in the field as biopesticide products result sometimes in inconsistent performance/efficacy, limiting their use^30^. It is therefore important to explore the effect of this key abiotic stress, temperature on the efficacy of the identified potent EPF isolates^18^ to sustainably manage *T. absoluta* in different agroecological systems, especially under the continuous climate change scenario. Furthermore, different nonlinear and linear models are used to estimate fungal growth over a wide range of temperature regimes and to predict the effect of EPF virulence in epizootic development among target insect pest populations^31,32^. Consequently, accurate prediction of the potential ecological fitness of these virulent fungal isolates is fundamental to optimize their efficacy in field application against the tomato leafminer.

Akutse et al.^18^ recently reported the efficacy of three *Metarhizium anisopliae* (Metchnikoff) Sorokin fungal strains (ICIPE 18, ICIPE 20 and ICIPE 665) as the most potent isolates which hold promise as biocontrol agents for managing adults *T. absoluta*. However, the interactions between their performance and abiotic factors that could affect their field efficacy are paramount to be established for an effective selection of the most virulent fungal isolate(s) best suited for mass-production prior to its/their formulations and field deployment. To achieve this, it is important to simulate the effects of different temperature regimes on *M. anisopliae* ICIPE 18, 20 and 665 mass-production and their efficacy/virulent against *T. asboluta*, and subsequently predict potential suitable areas of application of these biopesticides or strengthen their efficacy through appropriate new formulations. Therefore, this study aimed to (i) assess the germination, growth and conidial production of the three candidate isolates, (ii) evaluate their virulence against adult *T. absoluta* under different temperature regimes, (iii) determine mass-production indices for the three fungal isolates and (iv) develop spatial predictions on potential areas where the candidate fungal isolates could cause significant epizootics in *T. absoluta* populations.

## Results

### Effect of temperature on conidial germination

**Figure 1:**
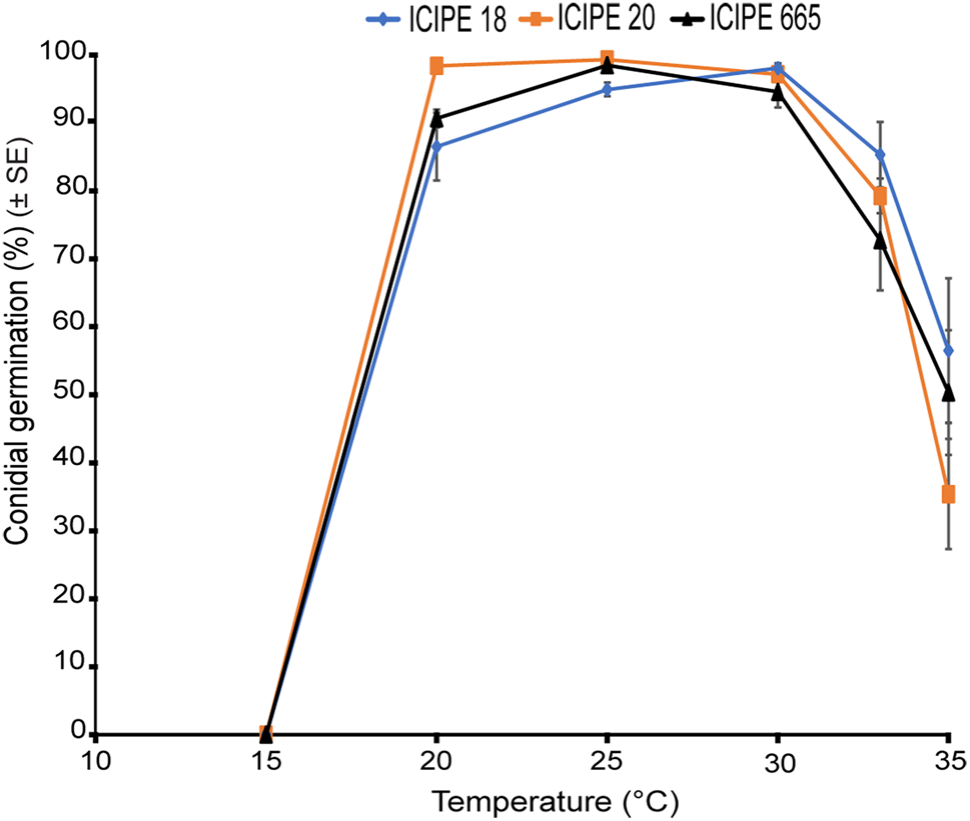
Effect of temperature on conidial germination of the three *Metarhizium anisopliae* fungal isolates ICIPE 18, 20 and 665. Error bars indicate the standard error of the mean at 95% CI (n = 72; N = 4; Tukey’s HSD test).

Conidia of the three *M. anisopliae* isolates germinated at all tested temperatures (ranging from 5 to 100%), except at 15 °C where no germination was observed 18 h post-incubation. Temperature significantly affected conidial germination (χ^2^ = 724.62; df = 5; *P* < 0.001) while fungal isolates did not have any effect on the germination (χ^2^ = 1.34; df = 2; *P* = 0.51) (Fig. 1). Significant differences in germination were observed at 20 °C (χ^2^ = 13.54; df = 2; *P* < 0.01) and 25 °C (χ^2^ = 14.51; df = 2; *P* < 0.001), with *M. anisopliae* ICIPE 20 achieving the highest germination rate. However, there were no significant differences among all the fungal isolates at 30 °C (χ^2^ = 3.30; df = 2; *P* = 0.19), 33 °C (χ^2^ = 2.89; df = 2; *P* = 0.24) and 35 °C (χ^2^ = 1.49; df = 2; *P* = 0.47). The optimum temperatures for conidial germination were observed at 20 °C, 25 °C and 30 °C for all the three isolates (Fig. 1). The interaction between fungal isolates and temperature significantly affected conidial germination (χ^2^ = 18.6; df = 10; *P* = 0.04).

### Effect of temperature on fungal growth

**Figure 2:**
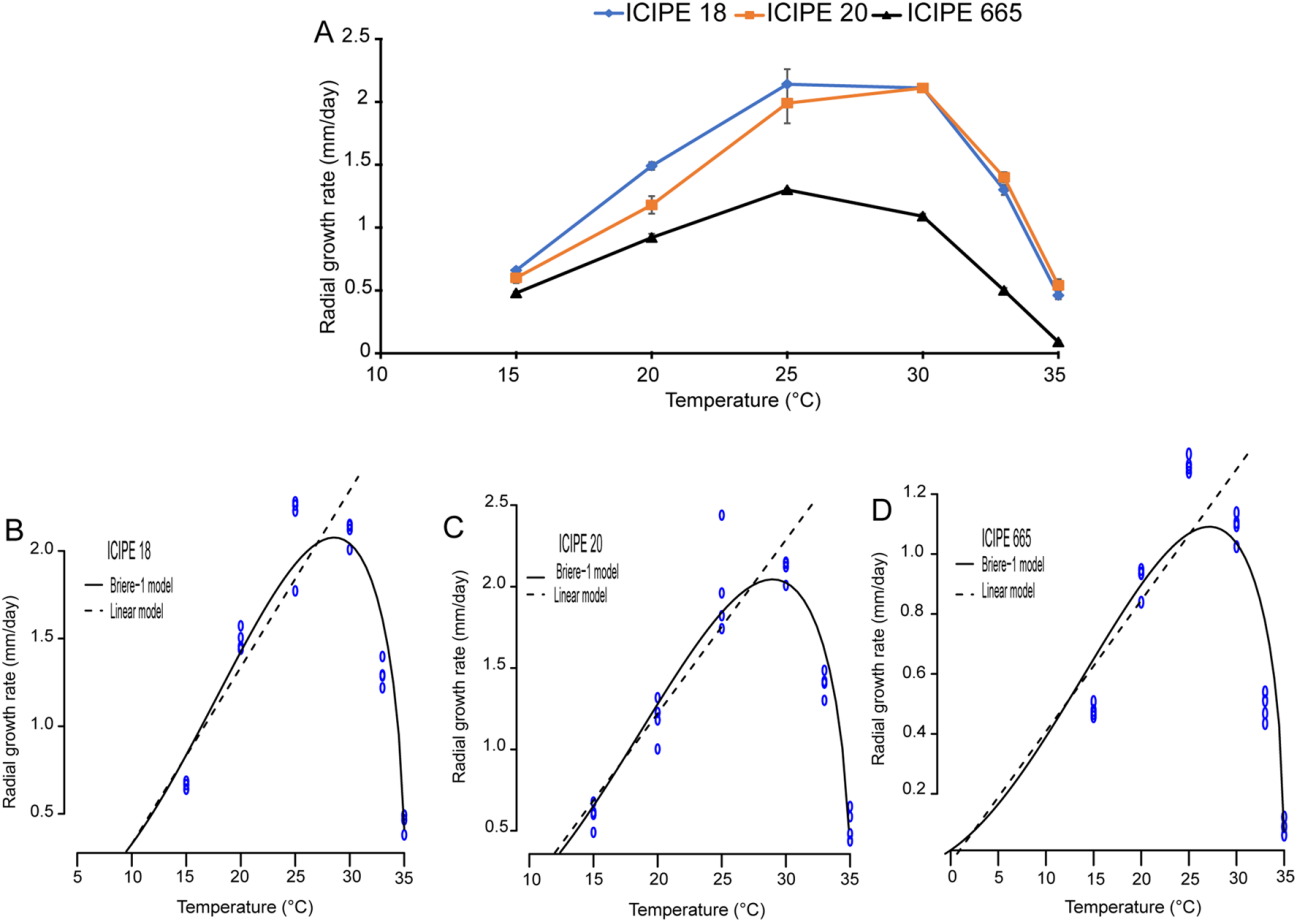
Relationship between temperature and radial growth rate of the three *Metarhizium anisopliae* fungal isolates. (**A**) Relative growth rates of *Metarhizium anisopliae* isolates ICIPE 18, ICIPE 20 and ICIPE 665 on SDA medium between 15 and 35 °C. Linear (dashed lines) and Brière-1 (continuous lines) nonlinear models fitted to observed values of the radial growth rate of *Metarhizium anisopliae* isolates (**B**) ICIPE 18, (**C**) ICIPE 20 and (**D**) ICIPE 665 at constant temperatures (n = 72; N = 4; Tukey’s HSD test).

The growth of the three isolates occurred at all temperatures, but was slower at 15, 33 and 35 °C as compared to 20, 25 and 30 °C (Fig. 2A). Temperature (F = 339.01; df = 5, 54; *P* < 0.001) and fungal isolates (F = 234.33; df = 2, 54; *P* < 0.001) significantly affected the mean radial growth. Mean radial growth differs significantly among the three fungal isolates at 15 °C (F = 14.57; df = 2, 9; *P* < 0.01), 20 °C (F = 42.33; df = 2, 9; *P* < 0.001), 25 °C (F = 15.28; df = 2, 9; *P* < 0.01), 30 °C (F = 369; df = 2, 9; *P* < 0.001), 33 °C (F = 224.8; df = 2, 9; *P* < 0.001) and 35 °C (F = 53.17; df = 2, 9; *P* < 0.001) (Fig. 2A). The highest growth rate occurred at 30 °C for both ICIPE 18 and ICIPE 20 and at 25 °C for ICIPE 665 (Fig. 2A). At all temperature regimes, isolates ICIPE 18 and ICIPE 20 grew faster than ICIPE 665 (Fig. 2A). Like the conidial germination, the interaction between temperature and fungal isolates significantly affected the radial growth (F = 13.43; df = 10, 54; *P* < 0.001). Parameter estimates obtained from the nonlinear Brière-1 model and linear models fitted to the radial growth rate are presented on Table 1 The fitted models for radial growth rate versus temperature for all the three fungal isolates are presented in Fig. 2B,C,D. The minimum temperature threshold (*T*_*min*_) estimated using the Brière-1 model was lower as compared to estimates of the linear regression model for the three fungal isolates (Table 1). The Brière-1 nonlinear model had a good fit to the data and predicted a lower temperature threshold of 4.45, 8.04 and −8.27 °C, for ICIPE 18, ICIPE 20 and ICIPE 665, respectively. The upper temperature threshold (*T*_*max*_) was 35.11, 35.16 and 35.01 °C, for ICIPE 18, ICIPE 20 and ICIPE 665, respectively with an optimum temperature of 29.86, 29.31 and 29.55 °C, for ICIPE 18, ICIPE 20 and ICIPE 665, respectively (Table 1). Using the linear model, *T*_*min*_ of the fungal isolates was estimated at 9.1 °C, 8.45° and 1.25 °C for ICIPE 18, ICIPE 20 and ICIPE 665, respectively. The optimum temperature threshold (*T*_*opt*_) was estimated at 29.86, 29.31 and 29.55 °C for ICIPE 18, ICIPE 20 and ICIPE 665, respectively (Table 1). The linear regression model showed a strong positive relationship between temperature and radial growth rate (*R*^2^ = 0.82 and *R*^2^ = 0.87 for ICIPE 18 and ICIPE 20, respectively) (Table 1).

**Table 1:** Estimated parameters and their approximative standard errors for linear and Brière-1 nonlinear model describing the relationship between temperature and growth of *Metarhizium ansiopliae* isolates.

Model	Parameters	Fungal isolates		
		ICIPE 18	ICIPE 20	ICIPE 665
Linear	a	−0.64 ± 0.28	−0.93 ± 0.24	−0.05 ± 0.19
	b	0.1 ± 0.01	0.11 ± 0.01	0.04 ± 0.01
	*T*_*min*_	9.1	8.45	1.25
	*R*^2^	0.82 ± 0.27	0.87 ± 0.23	0.64 ± 0.19
Brière-1	*T*_*min*_	4.45 ± 2.03	8.04 ± 1.35	−8.27 ± 8.15
	*T*_*max*_	35.11 ± 0.06	35.16 ± 0.06	35.01 ± 0.04
	*T*_*opt*_	29.86	29.31	29.55

### Effect of temperature on conidial production or sporulation

**Figure 3:**
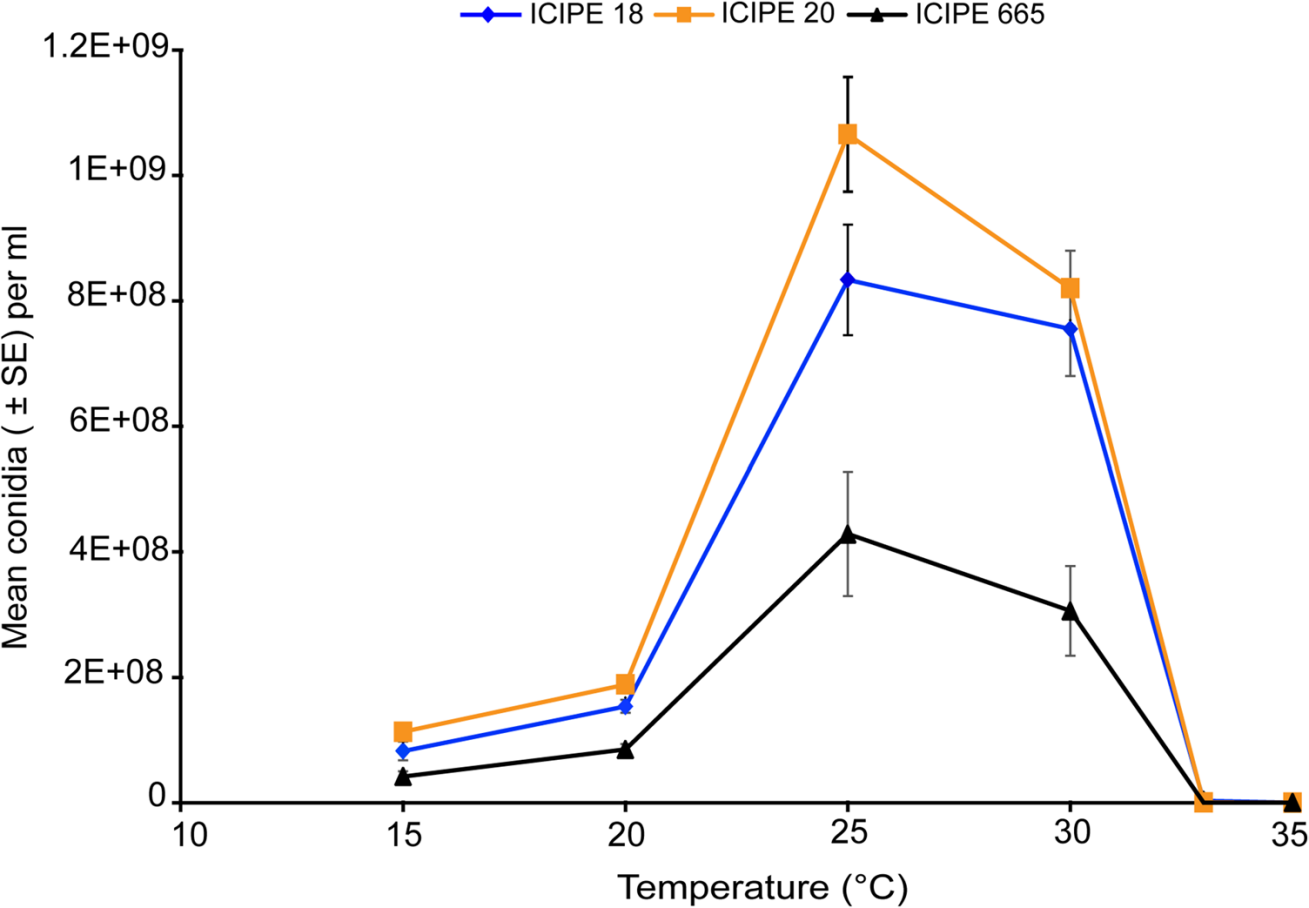
Effect of temperature on conidial production of the three *Metarhizium anisopliae* isolates ICIPE 18, 20 and 665. Error bars indicate the standard error of the mean at 95% CI (n = 72; N = 4; Tukey’s HSD test).

Conidial production varied from a low rate of 2.4 × 10^4^ conidia/ml (ICIPE 18, 35 °C) to a maximum of 1.06 × 10^8^ conidia/ml (ICIPE 20, 25 °C) (Fig. 3). Temperature (χ^2^ = 3195.5; df = 5; *P* < 0.001) and fungal isolates (χ^2^ = 69.4; df = 2; *P* < 0.0001) significantly affected conidial production. Also, the interaction between temperature and fungal isolates affected conidial yield (χ^2^ = 119.9; df = 10; *P* < 0.001). Isolates ICIPE 18 and ICIPE 20 yielded the highest conidial density at 15, 20, 25 and 30 °C. The best sporulation rate for all the three isolates was obtained at 25 °C (Fig. 3).

### Effect of temperature on the virulence of *Metarhizium anisopliae* fungal isolates against adults of the tomato leafminer *Tuta absoluta*

**Figure 4:**
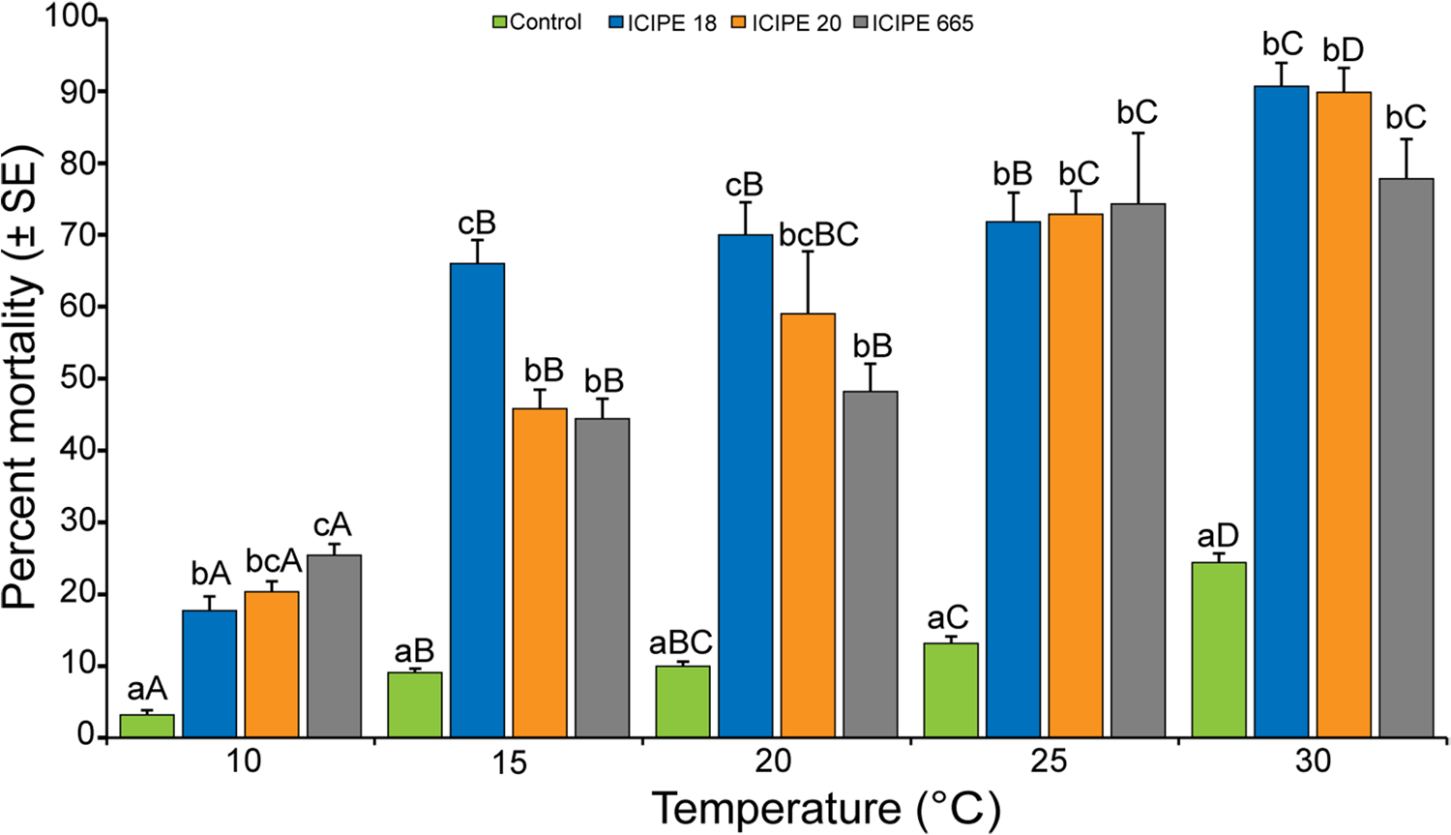
Effect of temperatures on virulence of the three isolates of *Metarhizium anisopliae* against adult *Tuta absoluta*, 12 days post-inoculation. Different lowercase letters show significant difference (GLM, *P* ≤ 0.05) in mortality among fungal isolates across the different temperature regimes. Different uppercase letters denote significant difference (GLM, *P* ≤ 0.05) in mortality across the different temperature regimes for each isolate (n = 60; N = 4; Tukey’s HSD test).

**Figure 5:**
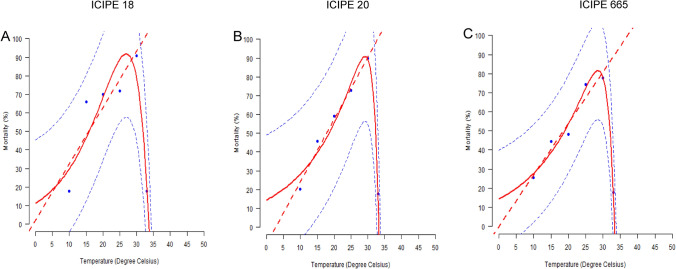
Observed and predicted mortality of adult *Tuta absoluta* by *Metarhizium anisopliae* isolates ICIPE 18, ICIPE 20 and ICIPE 665 in relation to temperature using the linear and nonlinear models. The blue dots on the graph represent the cumulative values of the proportion of adult *Tuta absoluta* that were killed by the three isolates during the experiments at the respective temperatures. The curve represents the Logan-4 and Logan-1 nonlinear models that best fits the experimental data points and were used to predict the level of efficacy of isolates (**A**) ICIPE 18, (**B**) ICIPE 20 and (**C**) ICIPE 665 against adult *Tuta absoluta*. Fitted models are the dashed straight lines for linear regression and solid lines for the Logan models. Dashed lines above and below represent the upper and lower 95% confidence interval (n = 60; N = 4; Tukey’s HSD test).

All the three isolates were virulent against adults *T. absoluta* variably, with percentage mortality ranging from 18–91%, 20–90% and 25–78% for ICIPE 18, ICIPE 20 and ICIPE 665, respectively across temperature regimes of 10–30 °C (Fig. 4). Mortality increased significantly (χ^2^ = 422.54, df = 4, *P* < 0.001) with increase in temperature. The highest mortality occurred at 30 °C for all the three fungal isolates. Isolates had significant effect (χ^2^ = 15.23, df = 2, *P* < 0.001) on adults *T. absoluta* mortality. At 10 °C ICIPE 665 caused the highest mortality rate while at 15 and 20 °C, ICIPE 18 caused the highest mortality followed by isolates ICIPE 20 and ICIPE 665 (Fig. 4). However, no significant difference in mortality was observed among the fungal isolates at 25 (χ^2^ = 0.18, df = 2, *P* = 0.91) and 30 °C (χ^2^ = 5.77, df = 2, *P* = 0.06). The interaction between temperature and fungal isolates significantly affected percentage mortality (χ^2^ = 45.96, df = 8, *P* < 0.001). The lethal time required for 50% of insects to die (LT_50_) was calculated for all the three isolates that caused > 50% mortality 12 days post-treatment (Table 2). The speed of infection is faster at 25 and 30 °C than at low temperatures (10 and 15 °C) for all the three isolates. The LT_50_ values of ICIPE 18 and ICIPE 20 were significantly lower at 25 and 30 °C compared to ICIPE 665 (at 25 °C F = 4.92; df = 2, 9, *P* = 0.036; and at 30 °C F = 5.13, df = 2, 9, *P* = 0.033) (Table 2). The Logan models had a good fit to the mortality data (Fig. 5). The Logan-4 nonlinear model gave the best fit to the data and predicted a minimum and maximum temperature threshold of 9.10 and 33.11 °C, respectively for ICIPE 18. The Logan-1 predicted a maximum temperature threshold (*T*_*max*_) of 33.27 and 33.36 °C for ICIPE 20 and ICIPE 665, respectively (Table 3).

**Table 2:** LT_50_ values 12 days post-exposure of adult *Tuta absoluta* to *Metarhizium anisopliae* fungal isolates dry conidia under different temperatures.

Temperature (°C)	LT_50_ ± SE (Days)		
	ICIPE 18	ICIPE 20	ICIPE 665
10	-	-	-
15	2.88 ± 1.19	-	-
20	3.59 ± 1.04 a	4.93 ± 0.93 a	3.21 ± 0.26 a
25	3.41 ± 0.97 ab	2.40 ± 0.47 b	6.43 ± 1.23 a
30	1.41 ± 0.13 b	1.48 ± 0.30 ab	2.92 ± 0.56 a

Means within a row followed by the same lower letter case are not significantly different (Tukey’s HSD test, *P* = 0.05). Dash (-) means the LT_50_ value was not estimated for cumulative mortality less than 50% at 12 days post-treatment.

**Table 3:** Model parameters of Logan models describing the effect of temperature on virulence of fungal isolates against adult *Tuta absoluta*.

Fungal isolates	Model		Parameters	*F* value	*df* 1, 2	*P* value	Adj *R*^2^	AIC
ICIPE 18	Logan-4	α	4119.195	9.11	5, 17	< 0.001	0.70	12.90
		k	1561.87					
		b	0.12					
		*T*_*min*_	9.10					
		*T*_*max*_	33.11					
		Dt	8.17					
ICIPE 20	Logan-1	Y	0.14	20.60	3, 5	0.04	0.92	−9.81
		*T*_*max*_	33.27					
		p	0.07					
		v	1.66					
ICIPE 665	Logan-1	Y	0.10	27.75	3, 5	0.03	0.94	−13.42
		*T*_*max*_	33.36					
		p	0.48					
		v	15.17					

*F*, *F*-test statistic; *df*, degree of freedom; *P*, probability value; *R*^2^, coefficient of determination; AIC, Akaike’s information criterion.

### Mass production indices

**Figure 6:**
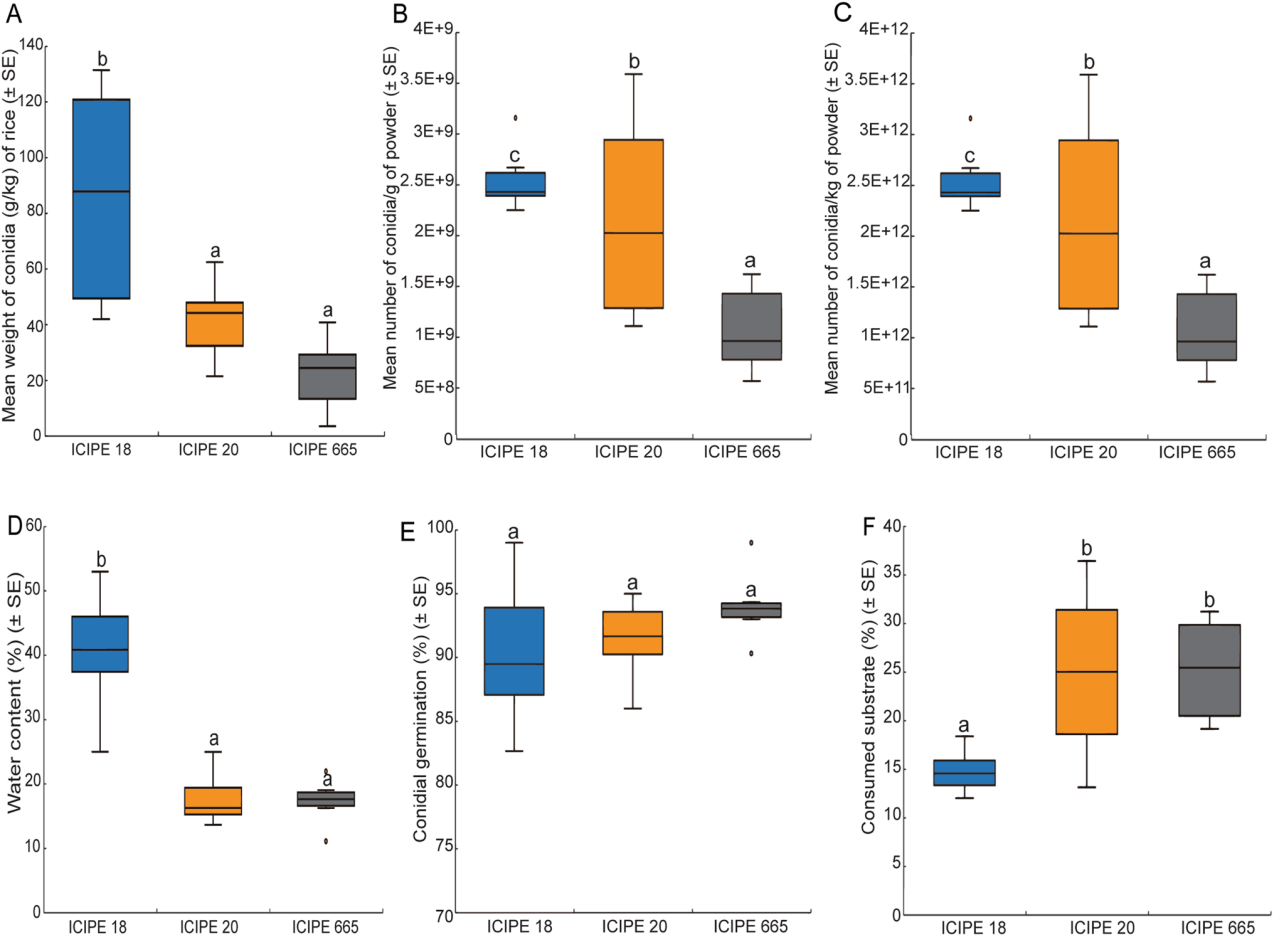
Mass-production indices of *Metarhizium anisopliae* isolates ICIPE 18, ICIPE 20 and ICIPE 665. (**A**) Mean weight of conidia powder/kg of rice. (**B**) Mean number of conidia/g of powder. (**C**) Mean number of conidia/kg of powder. (**D**) Water content (%) of conidia. (**E**) Percentage conidial germination and (**F**) Percentage consumed substrate. Different lowercase letters above error bars indicate a significant difference across the treatments (n = 18; N = 6; Tukey’s HSD test). Middle quartile (line that divides the box into two parts) shows midpoint of the data. Middle box represents 50% of the scores for each treatment and the middle 50% values fall within the inter-quartile range.

*Metarhizium anisolpiae* isolate ICIPE 18 had the most yield with a conidia powder weight of 86.30 ± 16.56 g/kg of rice compared to ICIPE 20 at 41.67 ± 5.95 g/kg and ICIPE 665 with 22.28 ± 5.54 g/kg (χ^2^ = 22.65, df = 2, *P* < 0.001) (Fig. 6A). In addition, both the number of conidia per gram of powder and the number of conidia per kg of powder were significantly higher for ICIPE 18 compared to ICIPE 20 and ICIPE 665 (conidia per gram: χ^2^ = 20.43, df = 2, *P* < 0.001; conidia per kg: χ^2^ = 20.48, df = 2, *P* < 0.001) (Fig. 6B,C). The percentage of water content was significantly higher for ICIPE 18 compared to ICIPE 20 and ICIPE 665 (F = 29.16, df = 2, *P* < 0.0001) (Fig. 6D). Regardless of the fungal isolates, the percentage of conidial viability was more than 90% and not significantly different among the fungal isolates (χ^2^ = 2.70, df = 2, *P* = 0.26) (Fig. 6E). The weight of the rice residues after conidia harvest was significantly lower (F = 7.13, df = 2, *P* < 0.01) in ICIPE 18 than in ICIPE 20 and ICIPE 665 (Fig. 6F).

### Spatial prediction of the virulence of *Metarhizium anisopliae* isolates ICIPE 18 and ICIPE 20 against adult *Tuta absoluta* in East Africa

**Figure 7:**
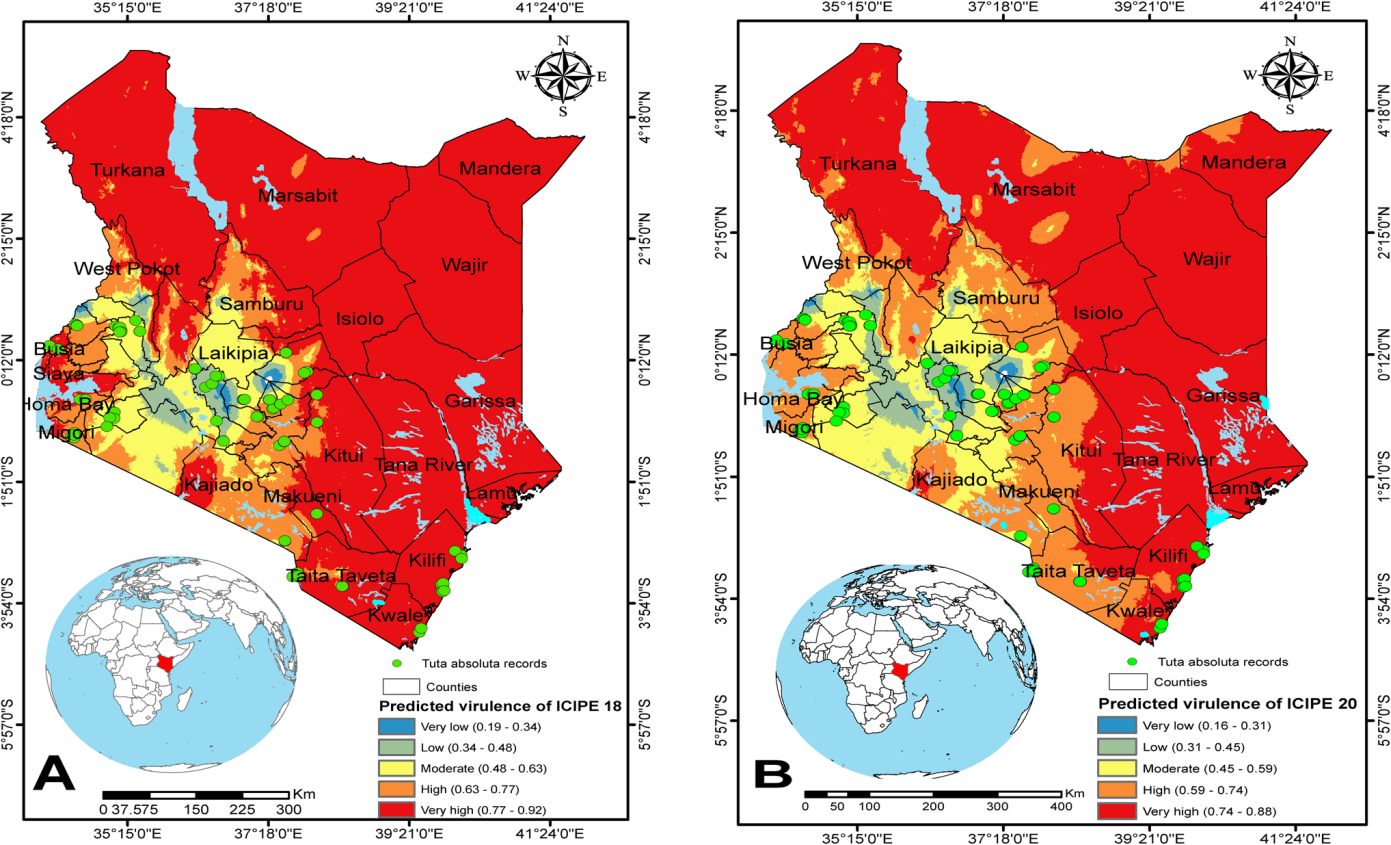
Spatial patterns of predicted virulence of *Metarhizium anisopliae* isolates ICIPE 18 and ICIPE 20 against adult *Tuta absoluta* in Kenya: (**A**) ICIPE 18 and (**B**) ICIPE 20. The dots in green indicate *Tuta absoluta* records in the three countries. The figures were generated using the QGIS 3.10.2 software (https://qgis.org/downloads/^33^).

**Figure 8:**
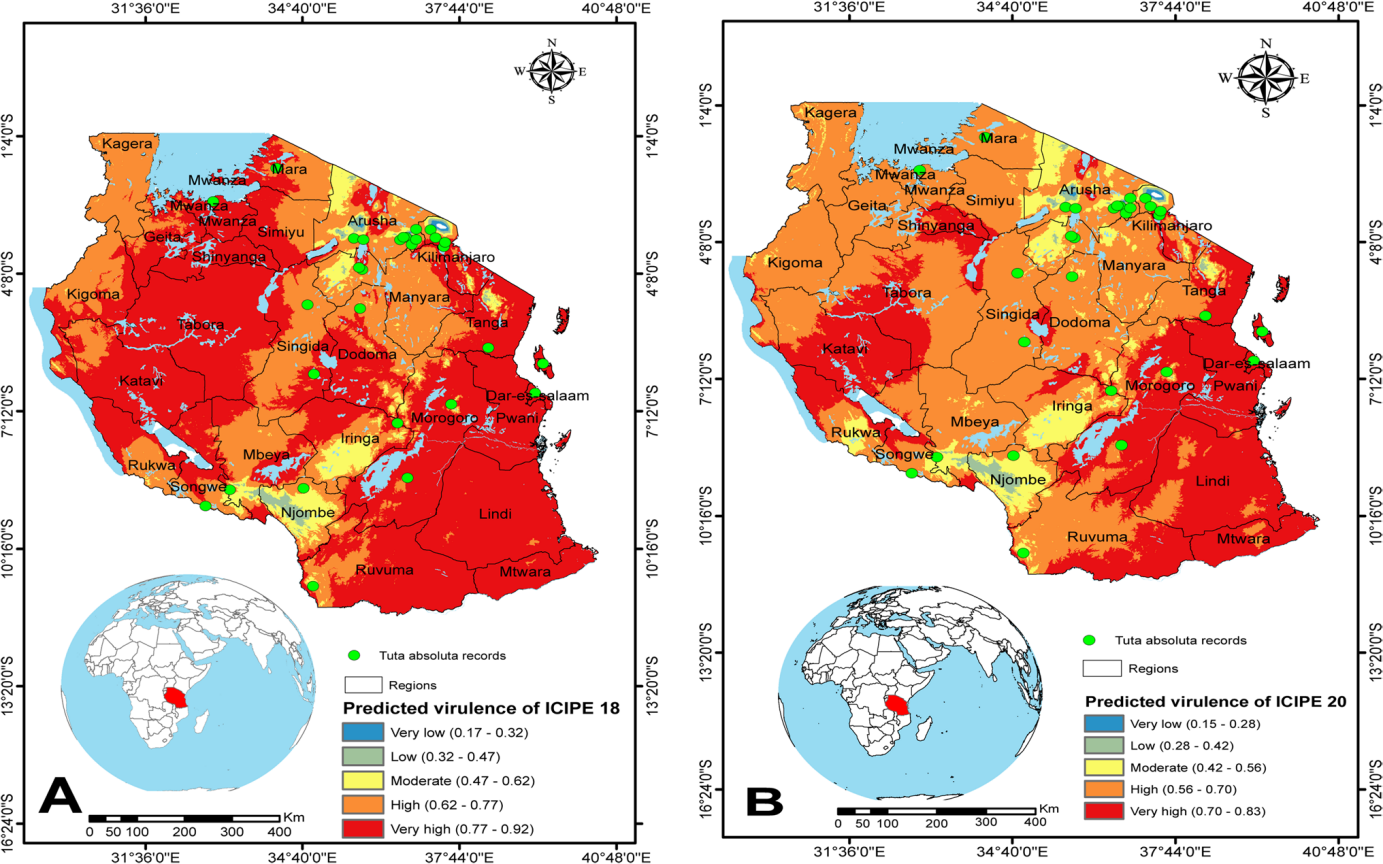
Spatial patterns of predicted virulence of *Metarhizium anisopliae* isolates ICIPE 18 and ICIPE 20 against adult *Tuta absoluta* in Tanzania: (A) ICIPE 18 and (B) ICIPE 20. The dots in green indicate *Tuta absoluta* records in the three countries. The figures were generated using the QGIS 3.10.2 software (https://qgis.org/downloads/^33^).

**Figure 9:**
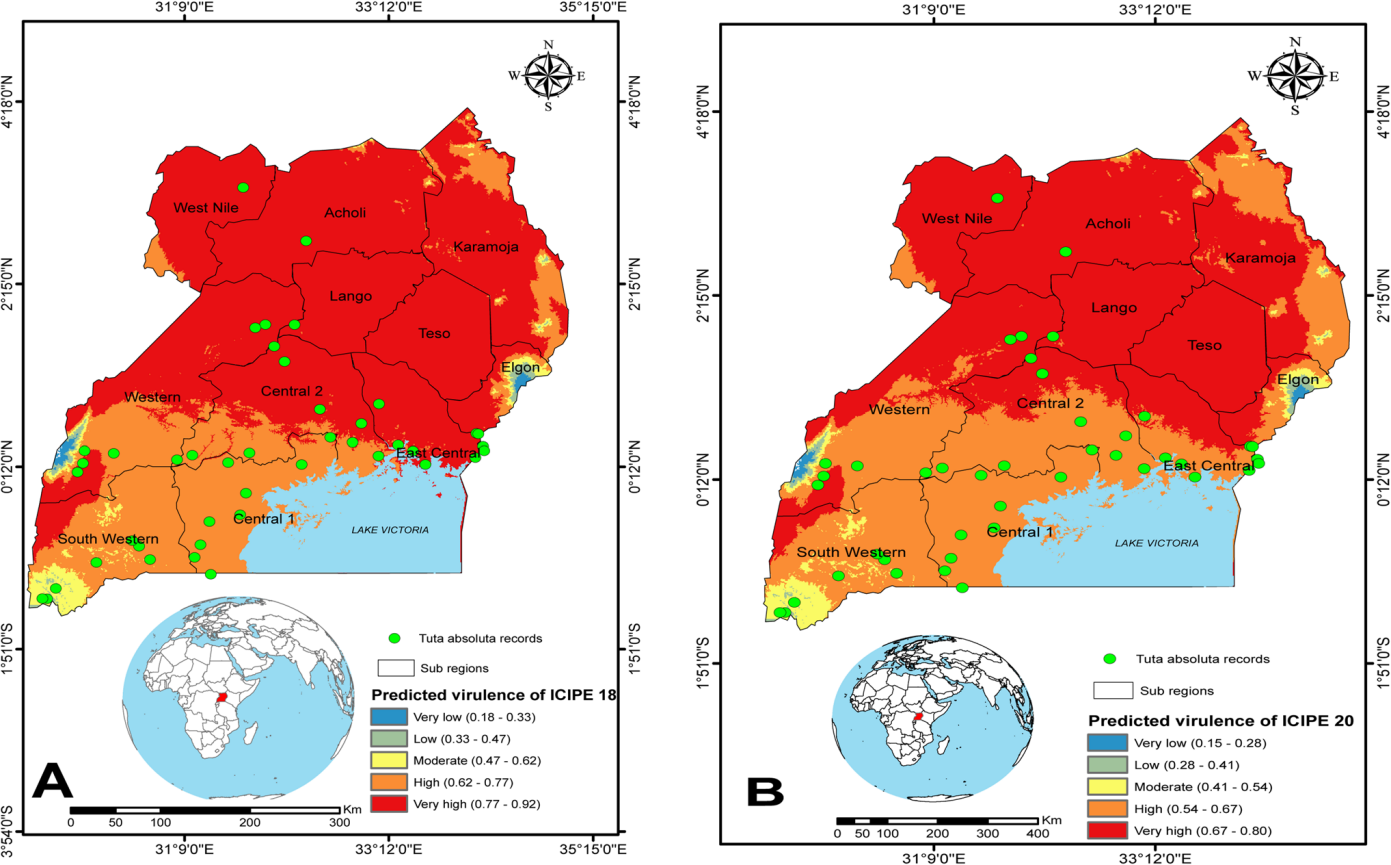
Spatial patterns of predicted virulence of *Metarhizium anisopliae* isolates ICIPE 18 and ICIPE 20 against adult *Tuta absoluta* in Uganda: (A) ICIPE 18 and (B) ICIPE 20. The dots in green indicate *Tuta absoluta* records in the three countries. The figures were generated using the QGIS 3.10.2 software (https://qgis.org/downloads/^33^).

Spatial predictions using EPFA for the performance or virulence of *M. anisopliae* isolates ICIPE 18 and ICIPE 20 against adult *T. absoluta* are shown in Figs. 7, 8 and 9. The pathogen performance model predicted that a deployment of *M. anisopliae* ICIPE 18 and ICIPE 20 in Kenya would result in high mortality (more than 70%) of adult *T. absoluta* in locations in the coastal part such as Taita Taveta, Kwale, Kilifi; and moderate mortality (45–63%) for locations in central Kenya such as Laikipia (Fig. 7A,B); unlike in some parts of Nakuru and Nyandarua where the model predicted very low performance of the fungal pathogen ranging from 16 to 34% mortality (Fig. 7A,B). Moreover, the model predicted a very high probability of mortality of adult *T. absoluta* in several regions in Tanzania (Lindi, Morogoro, Tabora, Tanga) for ICIPE 18 and a moderate mortality in Iringa and Njombe (Fig. 8A). Similarly, for ICIPE 20, the model predicted a high mortality pattern in regions of Singida, Dodoma, Mbeya, Manyara; but predicted moderate virulence pattern of ICIPE 20 in Iringa and Njombe (Fig. 8B). In Uganda, the virulence pattern was almost similar for the two isolates with high probability of mortality predicted in Lango, West Nile, Teso and Acholi; and very low to moderate mortality predicted in Elgon (Fig. 9A,B). In general, environmental conditions appeared to be conducive to the pathogens’ virulence across the three countries (Kenya, Tanzania and Uganda).

## Discussion

All the three *M. anisopliae* isolates (ICIPE 18, ICIPE 20 and ICIPE 665) tested in this study showed significant variation in germination, radial growth, conidial production and virulence against adult *T. absoluta* across the various temperature regimes with ICIPE 18 and ICIPE 20 showing their superiority as the best candidate biopesticides for sustainable management of the pest. Our results also showed that Kenya, Tanzania and Uganda where these potent isolates are expected to be registered, commercialized and upscaled for *T. absoluta* control exhibit different patterns in spatial virulence of the two candidate isolates (ICIPE 18 and ICIPE 20); which clearly demonstrates the importance of using spatial modelling as a decision-support tool for the optimization of biopesticides deployment in different agroecological zones. Furthermore, our study indicated that *M. anisolpiae* ICIPE 18 yielded the highest weight of conidia powder followed by ICIPE 20 and ICIPE 665 when using rice as growth substrate for mass-production of the candidate isolates.

The ability of an EPF isolate to germinate under given environmental temperature regimes is a critical determinant of its efficacy^34^. Here, our findings revealed that over 90% of conidia germinated at 20, 25 and 30 °C, while only between 35 and 50% germinated at 35 °C. At 15 °C, no spore germination was recorded after 18 h incubation, but low germination was observed after longer time (delayed germination) which consequently translates into low hyphal growth and low sporulation. This is in agreement with previous studies that revealed that no germination occurred at low temperatures (< 15 °C) for *M. anisopliae* fungal isolates^23,26^. Ekesi et al.^35^ also observed an absence or delayed conidial germination at low temperatures indicating that conditions were not favourable for spores to initiate germination within 18 h post-incubation. In contrast, Dimbi et al.^28^ recorded spores germination at 15 °C after 24 h inoculation for several *M. anisopliae* isolates including ICIPE 18 and ICIPE 20. Similarly, De Croos and Bidochka^36^ found that some *M. anisopliae* isolates were cold-active due to their ability to germinate and grow at temperatures as low as 8 °C. This suggests that spore germination may be related to the geographical origin of the strains or influenced by the conditions under which the spores are formed, highlighting the significant intra-specific variation in the germination among *M. anisolpliae* strains^36–39^. Importantly, germination occurred at 20 °C for all the three isolates; which marks the transition from a resting state to active development mostly driven by metabolic changes^40^. Our findings also showed that *M. anisopliae* isolates ICIPE 18 and ICIPE 20 had the highest germination rate at all temperature regimes compared to ICIPE 665 with an optimum at 25–30 °C. Hywel-Jones and Gillespie^38^ also reported an intra-specific variation in the germination among *M. anisopliae* strains with the highest germination rate achieved at 25 and 30 °C.

Growth of the three fungal isolates was adversely affected at 15, 33 and 35 °C. This finding concurs with previous studies that reported growth inhibition of fungal pathogens exposed to extreme temperatures^27,28^. The extremely low growth rate of the isolates observed at 15, 33 and 35 °C indicates that these temperatures were unsuitable for spores’ development and were close to the insect survival lower and upper thermal limits. Indeed, *T. absoluta* has a lower thermal threshold ranging from 5.37 to 7.38 °C while its upper thermal threshold varies between 33.82 to 35.69°C^41^. However, fungal growth for the three isolates became evident at 20 °C reaching an optimum at 30 °C predicted by the linear and Brière-1 models, even though ICIPE 665 grew more slowly than the other two isolates. This is in an agreement with Bayissa et al.^22^ and Ekesi et al.^34^ who reported that *M. ansiopliae* species are mesophilic fungi that grow well between 15 and 30 °C with an optimal temperature range of 20–30 °C. Vidal et al.^42^ reported similar growth pattern for *Paecilomyces fumosoroseus* fungal species exhibiting high growth rate between 8 and 30 °C with thermal optima ranging from 20 to 30 °C. It is important to note that for fungal isolates of tropical origin, growth does not occur below a lower thermal threshold (10 °C) and gradually the growth increases with increase in temperature to a maximum at optimal temperature (25–28 °C), and finally decreases rapidly to zero at an upper threshold (32–35 °C) which is considered as the lethal temperature^42,43^. Although the germination and fungal growth rate reached their optimum at 30 °C for isolates ICIPE 18 and ICIPE 20, we observed a significant decrease in spore production at the same temperature. Nevertheless, isolates ICIPE 18 and ICIPE 20 yielded the highest conidia production at all the temperature regimes compared to ICIPE 665 with an optimum at 25 °C. The poor yield of conidia observed at 33 and 35 °C illustrates the requirement/importance of optimum temperature to sustain conidia production and consequently boost fungal mass-production. This finding is consistent with the observation that optimum temperature for spore production is at 25°C^44^.

Adults *T. absoluta* exposed to dry conidia showed a sharp increase in mortality at temperatures between 10 and 30 °C. The highest level of infection was observed at 25 and 30 °C for all the three fungal isolates, presumably representing the optimum temperatures at which fungal infection is most affecting the insect. This virulence pattern observed is therefore important in the selection of key application zones of these fungal candidates since their virulence is showed to be temperature-dependent. The effect of temperature on virulence of *M. anisopliae* dry conidia against adult stages of insect pests has previously been reported by Onsongo et al.^27^ in adult Tephritid fruit flies *Zeugodacus cucurbitae* (Coquillet) (Diptera: Tephritidae) which succumbed to fungal infection over a wide temperature range (15–30 °C), with an optimum of 25 °C. A similar mortality pattern was observed with *M*. *anisopliae* isolate ICIPE 69 which was found effective against adult legume pod borer *Maruca vitrata* Fabricius (Lepidoptera: Crambidae) at temperature ranging from 15 to 33 °C, with an optimal temperature infection ranging between 25–30°C^26^. The lowest LT_50_ values were recorded at 25 and 30 °C for the most two virulent isolates, ICIPE 18 and ICIPE 20. The rapid germination and growth of these two fungal isolates at almost all the temperature range could probably explain the fastest death they induced to the insects observed in this study. This confirms the observation that fungal isolates tend to kill most rapidly at the optimum temperature of their vegetative growth^26^. These two candidate isolates could therefore be deployed in the field with different temperature variation (20–30 °C) using an autodissemination device against adult *T. absoluta* through an “attract-and-infect” strategy, as they were found to be compatible with the commercial *Tuta* pheromone lure (TUA-Optima®)^18^.

Mathematical models have become an important tool for understanding and predicting the virulence and suitable application areas of entomopathogenic fungi against insect pests of economic importance often occurring in unpredictable environmental conditions^31,45^. Here, we used *T. absoluta* occurrence in East Africa^4^ to model the virulence of the candidate isolates (ICIPE 18 and ICIPE 20) against the pest. The decision-support tool revealed that in some areas across Kenya, Tanzania and Uganda (where the potent fungal-based biopesticides are planned to be registered, commercialized and upscaled), the candidate fungal isolates might be effective as an excellent control and management tool for *T. absoluta* while in other areas the predicted level of virulence tend to be very low. For example, in the southern and northern part of Kenya, the models predicted higher virulence of both isolates while very low to moderate virulence has been predicted in the Central part of the country. Interestingly, tomato production is highly concentrated in Kirinyaga, Kajiado, Bungoma, Kwale and Taita Taveta counties^4^ where ICIPE 18 and ICIPE 20 are expected to perform by inducing high infection to the pest. In Tanzania, tomato is mainly cultivated in Iringa, Morogoro and Tanga regions^46^ while in Uganda, the main tomato production areas are Central, Eastern and Western regions^47^ where the model predicted high mortality pattern of the pest for the two candidate isolates. As such, deploying fungal-based biopesticides in these locations will have a high probability of managing *T. absoluta*. However the microclimate conditions in some counties of Kenya (e.g. Naivasha in Nakuru county) where tomato is produced in greenhouses could be suitable for the infection of the pest by the fungal isolates and this requires investigating further. Klass et al.^32^ predicted the effects of temperature on performance of a fungus-based biopesticide for controlling locusts and grasshoppers. The authors also predicted considerable spatial variation in *M. anisopliae* var. *acridum* and its virulence across different regions where the two voracious pests (locusts and grasshoppers) occur. These three countries (Kenya, Tanzania and Uganda) are among the countries where most biopesticides developed by *icipe* are also registered and commercialized. With a pest like *T. absoluta*, which is present permanently on tomato and other solanaceous fields, fungal isolates which can infect the pest under different agroecologies and remaining viable for an extended period would be more economical for smallholder farmers. Although these candidate biopesticides hold considerable promise, unpredictable weather conditions in the field could seriously undermine their performance leading to poor delivery. Therefore, the main challenge of predicting the spatial virulence of fungal pathogens lies in the high variability of the environmental factors for which temperature is not the only factor affecting the viability of biopesticides. Climatic factors like relative humidity (more specifically "vapor pressure deficit", VPD) which was not explored in this study have been shown to have great impacts on fungal growth and virulence. It is therefore important to assess the combined impact of temperature and relative humidity (VPD) in future studies.

One of the primary prerequisites for a pathogen to be developed as a biopesticide is its ease for mass-production^26,48^. We found that *M. anisopliae* ICIPE 18 outperformed the other two isolates ICIPE 20 and ICIPE 665, as it produced the highest conidial yield, consumed less substrate and displayed high moisture content indicating that this isolate is capable of colonizing the rice substrate and yields high conidia. This high sporulation capacity is an interesting feature which would definitely contribute to fast-track the registration of ICIPE 18 when field validation trials are conclusive. Interestingly, we recorded conidia yield higher than 2 × 10^9^ conidia/g of powder for both ICIPE 18 and ICIPE 20 which indicates that ICIPE 20 can equally grow and sporulate very well on rice substrate producing large number of infective spores therefore making it highly desirable for commercialization. Rice is considered as the most suitable substrate for fungal spores mass-production as it is locally available and provides a large surface area for sporulation^49^. Using rice as growth substrate, Barra et al.^50^ also reported a high production of conidia per gram (2.1 × 10^9^) for *Purpureocillium lilacinum* isolate JQ926212. Besides, we recorded a high level of moisture content (40%) for isolate ICIPE 18. Moisture plays a crucial role in conidial production of fungal isolates in solid-state fermentation as fungal spores require free moisture during the germination and host penetration process^51^.

In conclusion, *M. anisopliae* isolates ICIPE 18 and ICIPE 20 were found to be effective against adult *T. absoluta* and could be developed as biopesticides based on their efficacy across a broad range of temperature regimes (germination, growth and sporulation), speed of kill (LT_50_) and virulence against the pest. In addition, both isolates can successfully be mass-produced on rice using a simple, fast and cost-effective mass-production technique (especially for private sector for business incubation) suitable for deployment in the field. However, the successful deployment of these two biopesticides requires field validation trials under different agroecological zones for which the decision-support tool has provided us with tangible information related to the suitable locations where the two candidate fungal isolates are expected to cause significant epizootics in *T. absoluta* populations. Adequate fungal strains selection and their accurate spatial prediction are therefore fundamental approach to optimize their efficacy prior to field deployment and could consequently guide decision-making for private sector, farmer-based organizations and policy in promoting effective use of candidate fungal pathogens against adult *T. absoluta* in East Africa and beyond.

## Materials and methods

### Insects

Source colony of *T. absoluta* was initially established from wild adults and larvae collected from infested tomato leaves and fruits in Mwea (0°36′31.3″S 037°22′29.7″E), Kirinyaga county, Kenya in June 2019. The moths were kept in ventilated, sleeved Perspex cages (40 × 40 × 45 cm) and fed ad libitum with 10% honey solution placed on the top side of each cage^19^. Four potted tomato plants (*Solanum lycopersicum* L. cv. “Money maker” grown from seeds obtained from Simlaw Seeds Company Ltd., Nairobi, Kenya) were placed in the cages for oviposition. The plants were removed 24 h post-exposure to female insects and transferred to separate wooden cages (50 × 50 × 60 cm) with ventilated openings on both its sides and top covered with netting material until the eggs hatched. Leaves with larvae were removed from these plants, three days after the larvae hatched and placed into clean sterile plastic containers (21 cm long × 15 cm wide × 8 cm high) lined with paper towel to absorb excess moisture and fine netting infused lid for ventilation. The larvae were supplied daily with fresh tomato leaves as food until pupation. The pupae were collected from the plastic containers using a fine camel hair-brush and placed inside a clean plastic container (21 cm long × 15 cm wide × 8 cm high) for adult emergence. The colony was rejuvenated every three months through infusion, with infested tomato leaves collected from the field to reduce inbreeding^18,19^. Insects were maintained under a rearing condition of 28 ± 2 °C, 48% relative humidity (RH) and 12:12 L:D photoperiod at the Animal Rearing and Quarantine Unit (ARQU) of *icipe* for five generations prior to bioassays^19^.

### Fungal isolates and viability assessment

The three *M. anisopliae* fungal isolates (ICIPE 18, ICIPE 20 and ICIPE 665) used in this study were obtained from the International Centre of Insect Physiology and Ecology (*icipe*)’s Arthropod Pathology Unit Germplasm (Table 4). The isolates were cultured on Sabouraud dextrose agar (SDA) (OXOID CM0041, Oxoid Ltd., Basingstoke, UK), and maintained at 25 ± 2 °C in complete darkness. Conidia were harvested by scraping the surface of two- to three-week-old sporulated cultures using a sterile spatula. The harvested conidia were then suspended in 10 ml sterile distilled water containing 0.05% (w/v) Triton X-100 (MERCK KGaA, Darmstadt, Germany) and vortexed for five min at about 700 rpm to break conidial clumps and ensure a homogenous suspension. Conidial concentrations were quantified using an improved Neubauer hemocytometer under a light microscope^52^. The conidial suspension was adjusted to a concentration of 3 × 10^6^ conidia ml^−1^ through serial dilution.

**Table 4:** Source and identity of the three Metarhizium anisopliae isolates used in the study and their germination rates.

Fungal species	Isolates	Source	Locality/Country	Year of isolation	Germination rate (%)
*Metarhizium anisopliae*	ICIPE 18	Soil	Mbita (Kenya)	1989	97.3 ± 2.5
	ICIPE 20	Soil	Migori (Kenya)	1989	96.8 ± 1.4
	ICIPE 665	Soil	Kenya	2008	96.2 ± 0.8

Prior to commencement of the bioassays, spore viability was determined by plating evenly 0.1 ml of 3 × 10^6^ conidia ml^−1^ onto 9-cm Petri dishes containing SDA. Three sterile microscope cover slips (2 × 2 cm) were placed randomly on the surface of each inoculated plate. Plates were sealed with Parafilm membrane and incubated in complete darkness at 25 ± 2 °C and were examined after 16–20 h^52^. The percentage germination of conidia was determined from 100 randomly selected conidia on the surface area covered by each cover slip under a light microscope (400 ×) using the method described by Goettel and Inglis^52^. Conidia were considered to have germinated when the length of the germ tube was at least twice the diameter of the conidium^52^. Four replicates were made for each isolate making a total of 12 plates for all isolates.

### Effect of temperature on spore germination of *Metarhizium anisopliae* fungal isolates

Aliquots (0.1 ml) of a 3 × 10^6^ conidia/ml conidial suspension were spread with a sterile glass spreader over the surface of 9-cm Petri dishes containing SDA. Three sterile microscope cover slips were placed on each plate, and the plates securely sealed with Parafilm membrane as described above. The plates were then incubated in complete darkness at constant temperatures of 15, 20, 25, 30, 33 and 35 °C. At 18 h post-incubation, plates were flooded with lactophenol aniline cotton blue to halt germination and to stain the spores for easy visibility. Percentage germination of 400 conidia, as four randomly selected counts of 100 conidia, on each plate was assessed under the 400 × objective of a Leica DM500 light microscope (Leica Microsystems, Wetzlar, Germany) using the method described by Goettel and Inglis^52^. A conidium was considered to have germinated when the germ tube length was equal to or greater than the length of the conidia. Each plate served as a replicate with four replicates per fungal isolate making a total of 72 plates.

### Effect of temperature on radial growth and sporulation

Conidial suspensions of the three isolates (ICIPE 18, ICIPE 20 and ICIPE 665) were prepared from two-week-old sporulated cultures and adjusted at a concentration of 1 × 10^7^ conidia ml^−1^ prior to subculture. Aliquots (0.1 ml) were spread-plated on 9-cm Petri dishes containing SDA. Inoculated plates were then incubated in complete darkness at 25 °C for three days to obtain mycelial mats. Mycelial mats were cut from culture plates into round agar plugs using an eight-mm diameter cork-borer. Each agar plug (ca. five mm thick) was then transferred onto the center of a fresh SDA medium plate from which a similar size plug of media had been previously removed using the same cork-borer. The plates with implanted mycelial plugs were sealed with Parafilm membrane and incubated in complete darkness at 15, 20, 25, 30, 33 and 35 °C. Radial growth was recorded daily for 12 days using two cardinal diameters, through two orthogonal axes previously drawn on the bottom of each Petri dish to serve as a reference^53^. The experiment was replicated four times with each replicate originating from a different culture plate. Twelve days post-incubation, conidia were then harvested by scraping the surface of the sporulated cultures from each plate using a sterile spatula. The harvested conidia were then suspended in 10 ml sterile distilled water containing 0.05% Triton X-100 and vortexed for five min at about 700 rpm to break conidial clumps and ensure a homogenous suspension. Conidial concentrations were quantified using an improved Neubauer hemocytometer under a light microscope as described above^52^.

### Effect of temperature on the virulence of *Metarhizium anisopliae* fungal isolates against adults of the tomato leafminer *Tuta absoluta*

Temperatures above 30 °C were fatal to the insect given that all the adults in the control completely died during the first two days following their introduction into the incubator. Therefore, the bioassay was conducted at 10, 15, 20, 25 and 30 °C which are the representatives temperature range at which the pest occurs in the field^54^. Twenty one-day-old virgin (unmated newly emerged moths) *T. absoluta*, male and female (at the ratio of 1:1) were inoculated with dry conidia of the *M. anisopliae* isolates using velvet-coated plastic jars (60 × 40 cm) following the method described by Migiro et al.^55^ and Akutse et al.^18^. For each isolate, the device was contaminated with 0.3 g of dry conidia (equal to 0.15 × 10^9^ conidia/g), after which moths were introduced into the device for three minutes for them to pick up fungal spores. Evidence of infection was confirmed through visual observation of fungal spores strongly attached to the body of the insects. Control insects were exposed to fungus-free velvet plastic jars. Three (3) minutes post-exposure, contaminated insects were transferred into clean ventilated Perspex cages (15 × 15 × 15 cm) and provided with 10% honey solution placed on the top side of each cage as food source daily up to the end of the experiment. Each treatment consisted of 20 treated insects per replicate and incubated at 10, 15, 20, 25 and 30 °C with four replicates per isolate making a total of 1,200 insects. Mortality was recorded daily for 12 days. Dead insects were surface-sterilized using 1% sodium hypochlorite solution, then put in 70% alcohol for five seconds and followed by three rinses in sterile distilled water. Insects were placed in Petri dishes lined with moistened filter paper to promote fungal growth on the cadaver surface. Petri dishes were securely sealed with Parafilm membrane and incubated at 25 °C. Mortality as a result of fungal infection was confirmed by the presence of hyphae and conidia on the surface of the cadaver. Each treatment consisted of 10 treated insects per replicate and incubated at 25 °C with four replicates per isolate making a total of 120 insects.

### Conidia production, harvest and assessment of mass production indices

Dry aerial conidia of *M. ansiopliae* ICIPE 18, ICIPE 20 and ICIPE 665 were produced on long-grain rice substrate^26^. One kilogram of precooked parboiled rice substrate was transferred into breathable bio control PP bags (24 cm long × 14 cm wide) with double B filter (Unicorn Imp. & Mfg. Corp, Plano, Texas, US) and autoclaved at 121 °C for 60 min. After which the substrate was allowed to cool to 28 °C and inoculated with 50 ml of a three-day-old culture of blastospores/mycelia under a laminar flow cabinet. The bag was sealed under aseptic condition and incubated for 21 days at ambient conditions (26 ± 1 °C and 60–70% RH), after which the contents were transferred into sterile plastic buckets (33 × 25 × 13 cm) to allow the culture to dry for seven days at 26 ± 1 °C. Conidia were harvested by sifting through a mesh sieve (295 µm mesh size). Six replicated production sets were run for each isolate. At harvest, conidial powder from each bag and isolate was taken to estimate the following mass-production parameters: (i) weight of conidia powder per kg of rice, (ii) number of conidia per gram of powder, (iii) number of conidia per kg of powder, (iv) percentage water content (based on weight loss of 1 g powder dried at 120 °C for 2 h), (v) percentage viability based on counts of germinated conidia, and (vi) percentage consumed substrate in each bag (based on the weight of the dry rice substrate before inoculation and that of the dry substrate residues immediately after harvesting the conidia)^26^.

### Modelling the effect of temperature on the radial growth rate

For temperature-dependent models, both linear and nonlinear models were fitted to the calculated radial growth data. A linear function was fitted to the data to determine the relationship between growth rate and temperature^43^. The linear model expressed as y (t) = a + bt was used to estimate the relationship between relevant temperatures and growth rate of fungal isolates, where y is the rate of growth, t is ambient temperature, and intercept (a) and slope (b) as the model parameters. The minimum temperature threshold (*T*_*min*_) and standard error (*SE*_*Tmin*_) were calculated using Eqs. (1) and (2):

1 $${T}_{min}= \frac{-a}{b}$$

2 $${SE}_{{T}_{min}}= \frac{{y}_{m}}{b}\sqrt{\frac{{S}^{2}}{N \times {y}_{m}^{2}}+{\left[\frac{{SE}_{b}}{b}\right]}^{2}}$$

where y_m_ is the average value of the growth rate, b is estimated slope of fitted line, S^2^ is the residual mean square of the linear model, and N is the sample size^56^.

However, linear function cannot accurately capture the growth rate at extreme temperatures^43^. Many empirical nonlinear models such as Logan and Brière models are fitted to fungal growth rate^23,43^. This allowed determining the minimum temperature (*T*_*min*_), optimum temperature (*T*_*opt*_) and upper temperature (*T*_*max*_) thresholds. Optimum temperature threshold (*T*_*opt*_) is defined as the temperature when the fungal growth rate is observed to be maximal, while *T*_*max*_ is referred to threshold temperatures above which growth does not occur^43^. Among the nonlinear models evaluated, the nonlinear regression model of Brière-1 (Eq. 3) was fitted to the data so as to describe the fungal radial growth rate at the various temperature thresholds, *r*(*T*)^57^.

3 $$r\left(T\right)=n \times T \times \left(T-{T}_{min}\right) \times \sqrt{{T}_{max}- T}$$

where, *r* is considered as the radial growth rate, derived as a function of temperature *T*, n being an empirical constant, *T*_*min*_ being the lower developmental temperature threshold and *T*_*max*_ the upper temperature threshold. The optimum temperature (*T*_*opt*_) of the fungal growth was estimated using Eq. (4):^57^

4 $${T}_{opt}=\frac{2m{T}_{max}+\left(m+1\right){T}_{min}+ \sqrt{(4{m}^{2}{T}_{max}^{2}+{\left(m+1\right)}^{2}{T}_{min}^{2}-{4m}^{2}{T}_{min}{T}_{max}}}{4m+2}$$

where m is an empirical constant^57^.

### Goodness of fit and selection criteria of the model

The best-fitted model is selected based on the residuals and comparing Akaike’s Information Criterion (AIC) and the Model Selection Criterion (MSC). The accuracy of different linear models in fitting the data was determined by comparing the coefficients of determination (*R*^2^). Goodness-of-fit of the model was assessed using the coefficient of determination (for linear model; *R*^2^) or the coefficient for nonlinear regression (for nonlinear models; *R*^2^) and the residual sum of squares (RSS). Higher values of *R*^2^ and lower values for RSS suggest a better fit^58^. For the linear regression, the data points at 33 °C and 35 °C which deviated from the straight line through the other points were omitted for correct calculation of regression^23,43^.

### Modelling the effect of temperature on the virulence of fungal strains against adult *Tuta absoluta*

An open-source computer-aided tool built on R-codes and Java interface, the entomopathogenic fungi application (EPFA) software version 1.0^45^ was used for modelling the virulence of the fungal strains (ICIPE 18 and ICIPE 20) against adult *T. absoluta*. The recorded mortality was plotted against the corresponding temperature values from which nonlinear models were fitted function to the observed data^45^. The model parameters were estimated by fitting equations to the recorded mortality and the corresponding temperature values. Eighty two (82) models were fitted to the data and the best-fitted models were selected based on their coefficient of determination *R*^2^, adjusted *R*^2^, Akaike’s information criteria (AIC), the root-mean-squared error (RMSE) and residual sum of squares (RSS)^45^. In addition, nonlinear models allowed the assessment of the minimum temperature threshold (*T*_*min*_) and the maximum temperature threshold (*T*_*max*_). The Logan-4 model (Logan et al.^59^) predicted well the effect of temperature on virulence of ICIPE 18 against adult *T. absoluta* while the Logan-1 model gave the best fit to the virulence of ICIPE 20 and ICIPE 665. The mathematical expressions of the models are presented in Table 5.

**Table 5:** Mathematical equations describing the relationship between temperature and virulence of *Metarhizium anisopliae* fungal isolates.

Model	Equation	References
Logan-1	$m\left(T\right)=Y* \left({e}^{p*T}-{e}^{\left(p*{T}_{max}\frac{\left({T}_{max}-T\right)}{v}\right)}\right)$	Logan et al.^59^
Logan-4	$m\left(T\right)= \alpha \left(\frac{1}{1+k* {e}^{-b(T-{T}_{min})}}-{e}^{\frac{({T}_{max}-\left(T-{T}_{min}\right))}{Dt}}\right)$	

For Logan models α, Y, k, b, Dt, and v are the model parameters, *T*_*min*_ the minimum temperature threshold and *T*_*max*_ the upper temperature threshold (°C).

### Spatial prediction of the virulence of the most potent fungal isolates

*Metarhizium anisopliae* isolates (ICIPE 18 and ICIPE 20) were selected for the spatial prediction study based on germination/viability and growth patterns, the speed of kill (LT_50_ value) and the mortality rates they caused to adult *T*. *absoluta* across all tested temperature ranges. To predict the spatial virulence of each fungal isolate, the temperature-dependent mathematical expression obtained during the modelling step was run at each grid of the raster files of Kenya, Tanzania and Uganda using the monthly minimum and maximum temperature datasets obtained from WorldClim (http://www.worldclim.org/). The gridded temperature datasets were loaded into EPFA software, simultaneously extracted from the database and then organized in matrix format using longitude as column and latitude as a row^45^. A point object picks the temperature-dependent mathematical expression of the virulence for the isolates and this is consecutively applied in each geographical coordinate of the grid. The results were converted into ASCII file format (.asc) and transferred into an open source software Q-GIS^60^ for visualization^45^. The virulence map was produced for Kenya, Tanzania and Uganda after completing the fitting process.

### Data analyses

Conidial germination data were analyzed with generalized linear model (GLM) assuming a binomial distribution with the log link function. Percent mortality was corrected for control mortality using Abbott’s formula^61^. Mortality data were analyzed using logistic regression in a GLM for a binomial distribution using the logit link function. Time-mortality data were analyzed with GLM using the function “dose.p” from the MASS library, to generate LT_50_ estimates, along with slopes of the regression curves. GLM analysis was run for each replication, and the resultant LT_50_ values and their respective slopes were subjected to ANOVA to generate means. Additionally, data on sporulation (conidia production) and number of conidia per gram of powder were analyzed using GLM with negative binomial error distribution taking into account overdispersion. Data on weight of conidia powder per kg of rice were analyzed using GLM with gamma distribution. Percentage water content and percentage consumed substrate data were analyzed with beta regression. Whenever a significant difference was found, multiple means comparison was made using Tukey’s HSD *post-hoc* test to assess pairwise comparison, adjustment for LS means with α = 0.05.

All statistical analyses were performed using R (version 3.6.3) statistical software packages^62^ and all statistical results were considered significant at the confidence interval of 95% (*P* < 0.05).

### Ethics approval

The experimental research and field studies on plants, including the collection of plant material, complied with relevant institutional, national, and international guidelines and legislation. The appropriate permissions and/or licenses for collection of plant or seed specimens were obtained for the study. All insect rearing, handling and experiments were performed using standard operating procedures at the *icipe* Animal Rearing and Quarantine Unit as approved by the National Commission of Science, Technology and Innovations, Kenya (License No: NACOSTI/P/20/4253). This article does not contain any studies with human participants performed by any of the authors.

## Data Availability

The dataset generated during the current study are available from the corresponding author upon request.
